# Asiatic acid in anticancer effects: emerging roles and mechanisms

**DOI:** 10.3389/fphar.2025.1545654

**Published:** 2025-02-24

**Authors:** Rong Chen, Wan Zhang, Meizhi Zhang, Weidong Liu, Weike Feng, Yanan Zhang

**Affiliations:** ^1^ College of Traditional Chinese Medicine, Shandong University of Traditional Chinese Medicine, Jinan, Shandong, China; ^2^ Graduate School of Dalian Medical University, Dalian, Liaoning, China

**Keywords:** Asiatic acid, tumor, antitumor activity, tumor cells, invasion and metastasis

## Abstract

Medicinal plants are crucial in the comprehensive treatment of anti-tumor with the advantages of high efficacy, low toxicity, multiple pathways and multi-targets synergy, leading to be a focal point of study for many oncologists. Identifying effective monomer components with anti-tumor properties from medicinal plants has long been a crucial focus in the study and development of traditional Chinese medicine. This endeavor has significant research value and promising possibilities for further advancement. Asiatic Acid (AA), a pentacyclic triterpenoid derived from *Centella asiatica* (L.) Urb, is used in traditional Chinese medicine and has been shown to have anti-tumor properties on a range of tumor types. The present study assessed the anti-tumor properties of AA from five different perspectives: inhibiting proliferation, inducing apoptosis, inhibiting invasion and metastasis, regulating cell autophagy, enhancing the resistance of tumor cells to drugs, and minimizing adverse side effects.

## 1 Introduction

**FIGURE 1:**
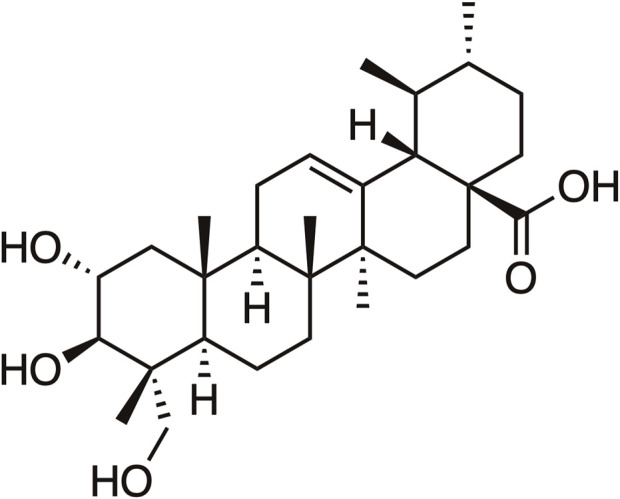
The chemical structure of Asiatic Acid.

In China, it was estimated about 4,824,700 new cancer cases and 2,574,200 new cancer deaths occurred in 2022, cancer remains a major public health concern in China (Han et al., 2024). Currently, the primary treatment choices for cancer patients consist of surgery, chemotherapy, or radiation. However, the efficacy of chemotherapy and radiation is limited by variables such as drug resistance and adverse side effects (Yan et al., 2017). Natural plants have a wide range of sources, minimal side effects, and inexpensiveness, discovering the active ingredients from them has been a crucial component in the development of anti-tumor traditional Chinese medicine (Zhou et al., 2022). Asiatic acid (AA), denoted by the molecular formula C_30_H_48_O_5_, is a pentacyclic triterpenoid constituent of the ursane-type skeleton that has been extracted from *Centella asiatica* (L.) Urb of the Apiaceae family (Its chemical structure of AA is shown in Figure 1). AA, the principal ingredient of *Centella asiatica*, is accountable for the *Centella asiatica’s* antitumor properties (Lin and Meng, 2021). Numerous studies have shown the various pharmacological effects of AA, including promoting wound healing (Diniz et al., 2023), anti-inflammation (Zou et al., 2022), hepatoprotective (Zhu et al., 2023), improving neurocognitive impairment (Ding et al., 2023), and reducing diabetic nephropathy (Ji et al., 2023). In addition, recent studies demonstrate that AA exhibits a wide range of anti-tumor characteristics and may effectively inhibit the growth of liver cancer cell, breast cancer cell, tongue squamous cell, prostate cancer cell, melanoma, and nasopharyngeal carcinoma cells (Islam et al., 2020; Pantia et al., 2023; Lai et al., 2023). This paper examines the anti-tumor properties of AA by analyzing its mechanisms and targets from five different perspectives: inhibiting cell proliferation, inducing apoptosis, preventing invasion and metastasis, regulating autophagy, enhancing drug resistance in tumor cells, and reducing side effects. The objective of this study is to assess the effectiveness of AA as a therapeutic agent against tumors.

## 2 Inhibition of cell proliferation by AA

Uncontrolled cell proliferation is a major characteristic of malignant tumors, and the ability to effectively suppress tumor cell proliferation is a crucial criterion for the advancement of anti-tumor drugs (Grody and W. Grody, 2016). Concerning the proliferation of human poorly differentiated hepatocellular carcinoma cells SMMC-7721, Wang et al. (2019) identified an inhibitory effect of AA that was dependent on concentration *in vitro*. Furthermore, AA was also found to significantly inhibit the growth of subcutaneous transplanted tumor of SMMC-7721 cells in nude mice *in vivo*. Chen Y. H. et al. (2023) verified that coadministration of AA with luteolin (Lut) could inhibit the cell cycle progression of cervical cancer CaSki and HeLa cells in the G1 phase, therefore significantly decreasing the viability of cervical cancer cells. Dong et al. (2019) reported that the proliferation of human tongue squamous carcinoma Tca8113 cells was inhibited by AA via upregulation of p53 and p21 protein expression and induction of cell cycle arrest at the G2/M phase in Tca8113 cells. Hao et al. (2018) carried out a study which presented empirical support for the notion that the introduction of AA into colon cancer SW480 and HTC166 cells substantially impeded their progression through the G2/M and S phases. Additionally, there was a substantial decrease in the levels of phosphorylated p-PI3K, p-AKT (Ser473), p-mTOR, and p-p70S6K. Conversely, there was an upregulation in the expression of the downstream programmed cell death factor 4 (Pdcd4). The anti-colon cancer effect of AA was evident via its regulation of the PI3K/AKT/mTOR/P70S6K signaling pathway. Guo et al. (2019) discovered that AA had the ability to hinder the transformation of hepatoma cells from G1 to G2/M phase in a way that depends on the dosage and duration, thereby blocking the proliferation of Huh7 cells. Concurrently, AA induction of p-p38 upregulation was observed in a dose-dependent manner, whereas p-AKT and p-ERK1/2 manifestation were diminished. These findings indicated that AA had the potential to suppress the proliferation of Huh7 cells via regulating the ERK and p38 signaling pathways.

**FIGURE 2:**
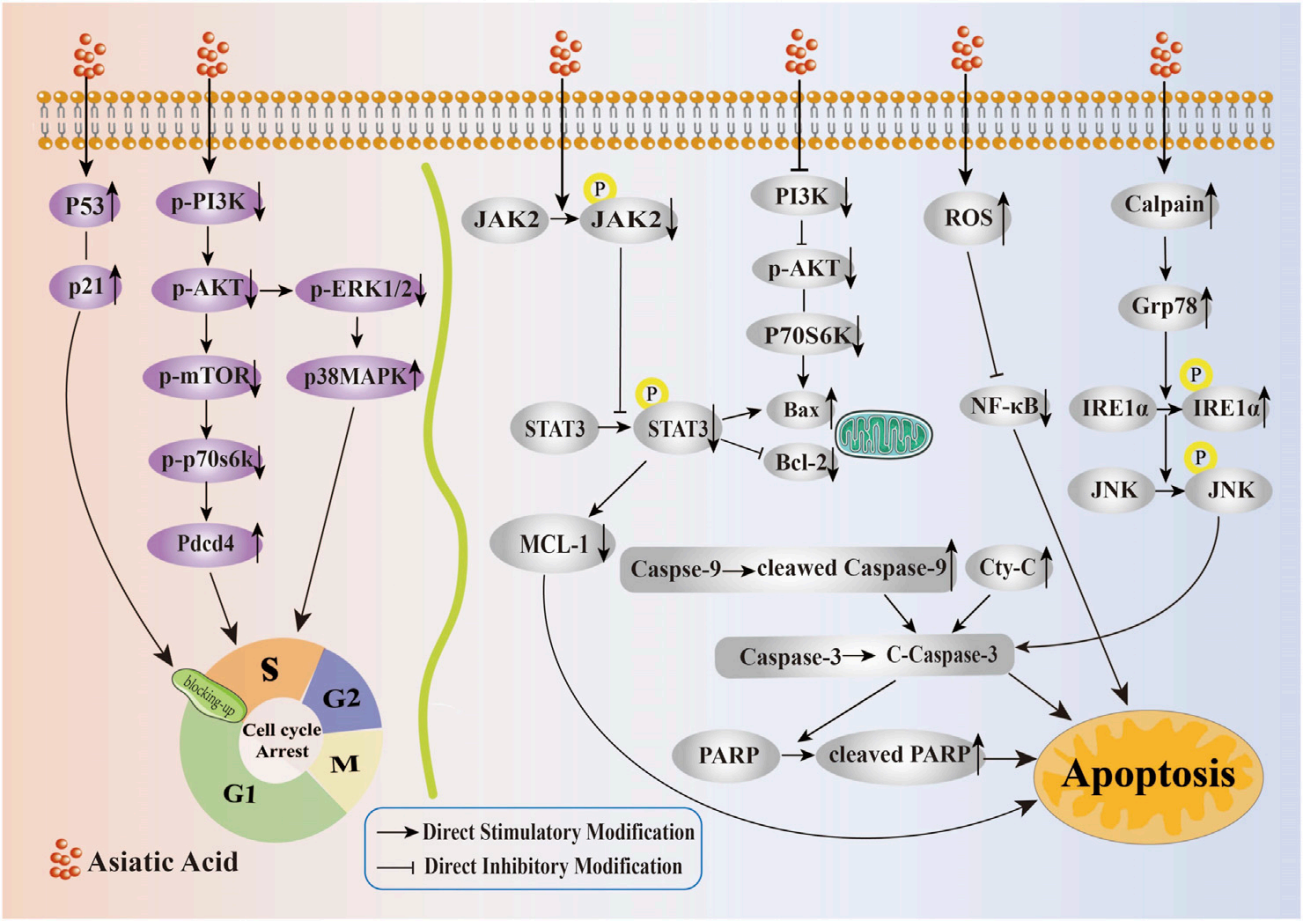
Mechanism of Asiatic Acid suppressing proliferation and inducing apoptosis in tumor cells. Asiatic Acid induces tumor cell cycle arrest by upregulating P53 and P21 protein expression, inhibiting PI3K/AKT/mTOR/P70S6K axis upregulation of Pdcd4 expression, downregulating p-ERK1, and increasing p-p38 expression. Asiatic Acid induces apoptosis of tumor cells involves multiple targets and pathways, such as mitochondrial apoptosis, endoplasmic reticulum stress induction, and triggering reactive oxygen species generation. Ultimately, it regulates the Bcl-2 family, drives Caspase-3 activation, and induces apoptotic cell death in tumor cells.

Overall, AA exerted a significant impact on the proliferation of tumor cells, primarily through the upregulation of P53 and P21 protein expression, inhibition of the PI3K/AKT/mTOR/P70S6K signaling pathway, promotion of the p38 signaling pathway, and inhibition of ERK, along with other multiple pathways to induce cell cycle arrest, thereby inhibiting the growth and proliferation of tumors (Its specific mechanism of inhibiting the proliferation is shown in Figure 2). Therefore, AA had significant potential for development as a therapeutic drug for cancer treatment.

## 3 AA inducing tumor cell apoptosis

Escaping apoptosis of tumor cells caused by various factors is crucial for the advancement of malignant tumors. Therefore, the capacity to restore or promote apoptosis in tumor cells is acknowledged as an effective strategy to eliminate tumor cells, which is also a crucial mechanism for most drugs, such as chemotherapeutic drugs, in controlling tumors (Carneiro and El-Deiry, 2020).

### 3.1 Bcl-2 gene family and caspase family

Liu et al. (2023a) discovered that AA significantly increased the Bax/Bcl-2 ratio and expression of BCL-2-associated X protein (Bax), cleaved Caspase-3, cytochrome C (Cyt-C) in prostate cancer DU145 cells, while downregulating the Bcl-2 protein expression. Furthermore, it was also possible that AA caused DU145 cells to undergo apoptosis by means of the mitochondrial route, as shown by the activation of Caspase-3 in DU145 cells. Their other study (Liu et al., 2023b) revealed that AA concentration-dependently increased the expression of Bax and cleaved Caspase-3 in prostate cancer PC-3 cells, downregulated the Bcl-2 expression of, facilitated the reduction of mitochondrial membrane potential leading to apoptosis of prostate cancer PC-3 cells, and could effectively inhibit the phosphorylation levels of JAK2 and STAT3 in the JAK2/STAT3 signaling pathway, thereby exerting an anti-prostate cancer effect. The expression levels of Cyt-C, cleaved Caspase-3, Bax, cleaved PARP, and cleaved Caspase-9 in human osteosarcoma HOS cells increased following treatment with AA, whereas the expression level of Bcl-2 decreased, according to a study by Yan et al. (2021). At the same time, there was a significant increase in the Bax/Bcl-2 ratio and Caspase-3 activity. In addition, the levels of myeloid cell leukemia sequence 1 (MCL-1), p-STAT3, and p-JAK2 expression in human osteosarcoma HOS cells were observed to significantly decrease in a way that depended on both time and concentration when exposed to AA. This finding suggested that AA potentially activated apoptosis of HOS cells via the mitochondrial pathway through the inhibition of MCL-1 and JAK2/STAT3 signaling. Furthermore, Chen Y. H. et al. (2023) discovered that the combination of AA and Lut resulted in a substantial upregulation of cleaved-PARP-1, Bax and cleaved Caspase-3, while concurrently downregulating the expression of Bcl-2 in cervical cancer CaSki and HeLa cells, thereby inducing caspase-mediated intrinsic apoptosis, Moreover, combination treatment with Lut and AsA more significantly decreased the PI3K(p110α), p-AKT, p-p70S6K, p-p38 and p-JNK1/2 expressions, and more synergistically increased the p-ERK1/2 expression level in both CaSki and HeLa Cells. The results indicated that the combined effect of LUT and AA involved the inactivation and activation of multiple signaling targets, and induced apoptosis by downregulating PI3K/AKT (PI3K, AKT and p70S6K) and JNK/p38 MAPK signaling pathways, upregulating the ERK signaling pathway, resulting in an anticancer effect on cervical cancer.

### 3.2 Triggering reactive oxygen species (ROS) generation

According to a study by Du et al. (2020), an increase in AA concentration led to a rise in reactive oxygen species (ROS) levels, while reduced glutathione (GSH) levels decreased in gastric cancer BGC-823 and HGC-27 cells. Simultaneously, there was a significant increase in the cellular expression of cleaved Caspase-3, cleaved Caspase-9, and cleaved PARP. AA upregulated the expression of Cyt-C in the cytoplasm and downregulated its expression in the mitochondria. The aforementioned findings suggested that AA inhibited the proliferation of gastric cancer cells via promoting ROS-mediated mitochondrial apoptosis. The activation of NF-κB plays a central role in the development of inflammation and cancer, the downregulation of NF-κB may be an exciting target in prevention and treatment of cancer (Khan et al., 2020). Alafnan et al. (2021) revealed that by elevating ROS levels, AA induced cell apoptosis in prostate cancer PC-3 cells by increasing Caspase activity and decreasing NF-κB expression. In the study conducted by Yang et al. (2022), they observed that AA intervention significantly increased ROS levels in breast cancer 4T1 cells, decreased mitochondrial membrane potential, upregulated cytoplasmic protein expression of Cyt-C, Bax, and cleaved-Caspase3, and downregulated Bcl-2 protein expression. This suggested that AA could promote apoptosis of breast cancer 4T1 cells via upregulating reactive oxygen species (ROS) production.

### 3.3 Endoplasmic reticulum (ER)

AA inhibited the growth of tongue squamous carcinoma cell line Tca8113 cells under endoplasmic reticulum (ER) stress conditions and induced the apoptotic death of these cells in a time-dependent fashion, Li et al. (2021) observed that an increase in the levels of Bax and cleaved Caspase-3 was accompanied by a decrease in the level of Bcl-2, Its mechanisms were related to the ability of AA to increase in calcium levels and activation of calcium-dependent protease (calpain), the release of calcium ions from the ER into the cytoplasm was a hallmark of ER stress, when cells were faced with ER stress, however,the accumulation of unfolded proteins results in glucose regulated protein 78kD Grp78 dissociation from IRE1α, increased expression of GRP78, which triggers the phosphorylation of Grp78-related IRE1α and JNK. Ultimately, this cascade of events activated Caspase-3 and triggers apoptotic cell death. The results of this study indicated that AA activated the ER Grp78/IRE1α/JNK signaling pathway, thereby inducing apoptosis in Tca8113 cells.

According to these findings, AA triggered apoptosis in tumor cells via the mitochondrial apoptotic pathway mediated by the Bcl-2 gene family and Caspase family, the ER stress-induced apoptosis pathway, the induction of ROS production, and activation of additional signaling pathways (Its specific mechanism of inducing apoptosis is shown in Figure 2).

## 4 Inhibiting tumor cell invasion and metastasis by AA

The process by which epithelial cells acquire the characteristics of mesenchymal cells is known as epithelial-mesenchymal transition (EMT) (Mittal, 2018). This transition is vital to the progression of malignancies because it facilitates cell migration, invasion, and infiltration (Wang et al., 2020). Alterations in associated biomarkers accompany the development of EMT, these alterations primarily consist of heightened expressions of zinc finger transcription factor (Snail), N-Cadherin, Vimentin, and matrix metalloproteinases (MMPs), while E-Cadherin and Claudin-1 exhibit diminished or absent expression (Lu et al., 2023; Chang et al., 2022)^.^

### 4.1 Wnt/β-catenin signaling pathway

Chen et al. (2022a) confirmed that AA inhibited MMP-9 and MMP-2 expression in colon cancer HCT116 cell. Additionally, it was shown that AA had a dose-dependent effect on decreasing the expression of N-cadherin, Vimentin, and snail, while simultaneously increasing the expression of E-cadherin. As a result, AA effectively hindered the process of cell EMT. This study demonstrated that AA decreased the expression of proteins related with the Wnt/β-catenin signaling pathway, including survivin, cyclinD1, β-catenin, and c-myc, in HCT116 cells. It is suggested that the mechanisms of AA’s inhibitory effect on HCT116 cell EMT was attributed to the suppression of the Wnt/β-catenin signaling pathway. Additionally, they found that Claudin-1 facilitated the migration and invasion of nasopharyngeal cancer cells through activation of the Wnt/β-catenin signaling pathway (Wu et al., 2018). Claudin-1 was linked to lymph node metastases and the clinical stage of nasopharyngeal carcinoma, according to Pantia et al. (2023) observed that AA demonstrated a considerable reduction in Claudin-1, N-cadherin, and β-catenin expression, an effective inhibition of STAT3 phosphorylation in nasopharyngeal carcinoma cells as well as an effective inhibition of STAT3 phosphorylation in nasopharyngeal carcinoma cells.

### 4.2 TGF-β signaling pathway

The impact of AA on the migratory and invasive capabilities of prostate cancer DU145 cells was investigated in the study by Liu et al. (2022). They discovered that AA effectively downregulated vascular endothelial growth factor A (VEGFA), fibronectin, Vimentin, N-Cadherin, and Snail proteins expression in DU145 cells induced by transforming growth factor β1 (TGF-β1), and upregulated the expression of E-Cadherin protein, leading to a reversal of the EMT process. It played a role in anti-invasion and metastasis of prostate cancer cells. Lewis mouse models of lung cancer and melanoma B16F10 were found to be inhibited by AA, according to Lian et al. (2021). In the tumor microenvironment, TGF-β/Smad signaling pathway was unbalanced, and the overactivation of Smad3 and the impairment of Smad7 promoted the invasion and metastasis of MMP-2-dependent cancers. AA served as a Smad7 enhancer, whereas naringenin (NG) inhibited Smad3. The combined treatment of AA and NG enhanced Smad7 expression, decreased the activity of Smad3, effectively reestablished the equilibrium of the smad3/Smad7 signaling pathway, thereby suppressing MMP-2 and increasing tissue metalloproteinase inhibitors. Therefore, AA effectively inhibited the invasion and metastasis of melanoma and lung cancer that were induced by TGF-β1 via its dependence on MMP-2.

### 4.3 PI3K/AKTs signaling pathway

Although it has been mentioned in Section 1 that AA could inhibit tumor cell proliferation through the PI3K/AKTs signaling pathway, its role in inhibiting tumor cell invasion and metastasis appears to be of more concern. Gou et al. (2020) observed that varying concentrations of AA affected the invasion capability of breast cancer MDA-MB-231 cells. Protein expression levels such as those of WAVE3, P53, NF-κB (P65), p-PI3K, and p-AKT were significantly diminished by AA. This implied that by blocking WAVE3 activation via the PI3K/AKT signaling pathway, AA prevented the migration of breast cancer cells. In their study, Wang et al. (2021) determined that AA inhibited the EMT process of non-small cell lung cancer A549 cells through the following mechanisms: reduction in migration capability, substantial downregulation of vascular endothelial growth factor (VEGF), MMP-14, and Vimentin protein expression, and inhibition of p-PI3K, p-AKT, and p-mTOR protein expression. It is suggested that a potential mechanism by which AA could impede the migration of A549 cells is through the PI3K/AKT/mTOR signaling pathway. Law et al. (2024) showed that AA suppressed the migration and invasion of osteosarcoma cells by downregulating MMP-1, and inhibited the phosphorylation of p-AKT as well, leading to a reduction in the specific protein 1 (Sp1) expression. The inclusion of a PI3K inhibitor augmented the effect of AA, suggesting that AA inhibited the migration and invasion of osteosarcoma cells via the AKT/Sp1/MMP 1 pathway.

### 4.4 p38MAPK signaling pathway

It was observed by Huang et al. (2022) that AA exhibited substantial inhibitory effects on the invasion and migration of RCC cells. This effect was likely due to its ability to inhibit the p-ERK/p-p38MAPK pathway and downregulate the expression of MMP-15. In their investigation, Lai et al. (2023) shown that AA inhibited the metastasis of prostate cancer PCa cells by blocking the transcriptional activity of Snail. This was due to AA disrupting the binding of the myeloid zinc finger1 (MZF-1) and ETS Transcription Factor ELK1 (Elk-1) proteins, impairing the ability of the complex to bind to the Snail promoter region, and inhibiting the phosphorylation of MEK3/6 p38MAPK. By inhibiting the MEK3/6-p38/MAPK signaling transduction pathway, it was hypothesized that AA could influence the MZF-1/Elk-1/Snail signaling axis to impede the migration and invasion of PCa cells.

### 4.5 FAK signaling pathway

Dong (2021) discovered that AA effectively suppressed the process of EMT in tongue squamous carcinoma Tca8113 cell by increasing E-cadherin expression and decreasing N-cadherin and Vimentin expression. Additionally, the study revealed that AA upregulated p53 protein expression and downregulated focal adhesion kinase (FAK) protein expression. These findings suggested that AA impeded the invasion and migration of Tca8113 cells by blocking EMT of Tca8113 cells through p53/FAK signal transduction pathway.

### 4.6 VEGF signaling pathway

Chen J. Y. et al. (2023) utilized mice with ascitic carcinoma S180 tumors to investigate the antitumor effects caused by AA. They discovered that AA had a significant effect on reducing the expression levels of heat shock protein 90 (Hsp90), which was associated with cell proliferation. Additionally, AA also reduced the levels of vascular endothelial growth factor (VEGF-C), a protein related to lymphatic vessels, and lymphatic endothelial hyaluronic acid receptor 1 (LYVE-1) in the mice’s tumors. Therefore, it was evident that AA exerted its influence on tumor growth inhibition and metastasis prevention through the regulation of Hsp90, LYVE-1, and VEGF-C protein expression. Tian et al. (2021) demonstrated that AA had considerable effects on breast cancer 4T1 tumors. It effectively decreased the density of microvessels and the permeability of blood vessels by suppressing the production of VEGF in human umbilical vein endothelial cells (HUVECs). Subsequently, the phosphorylation of vascular endothelial growth factor receptor 2 (VEGFR2) and its downstream target proteins, Src, FAK, and ERK1/2, were downregulated. These results suggested that through specific targeting of the VEGF/VEGFR2 signaling pathway, AA could effectively impede angiogenesis and vascular permeability. Consequently, the proliferation and metastasis of 4T1 cells were effectively suppressed.

**FIGURE 3:**
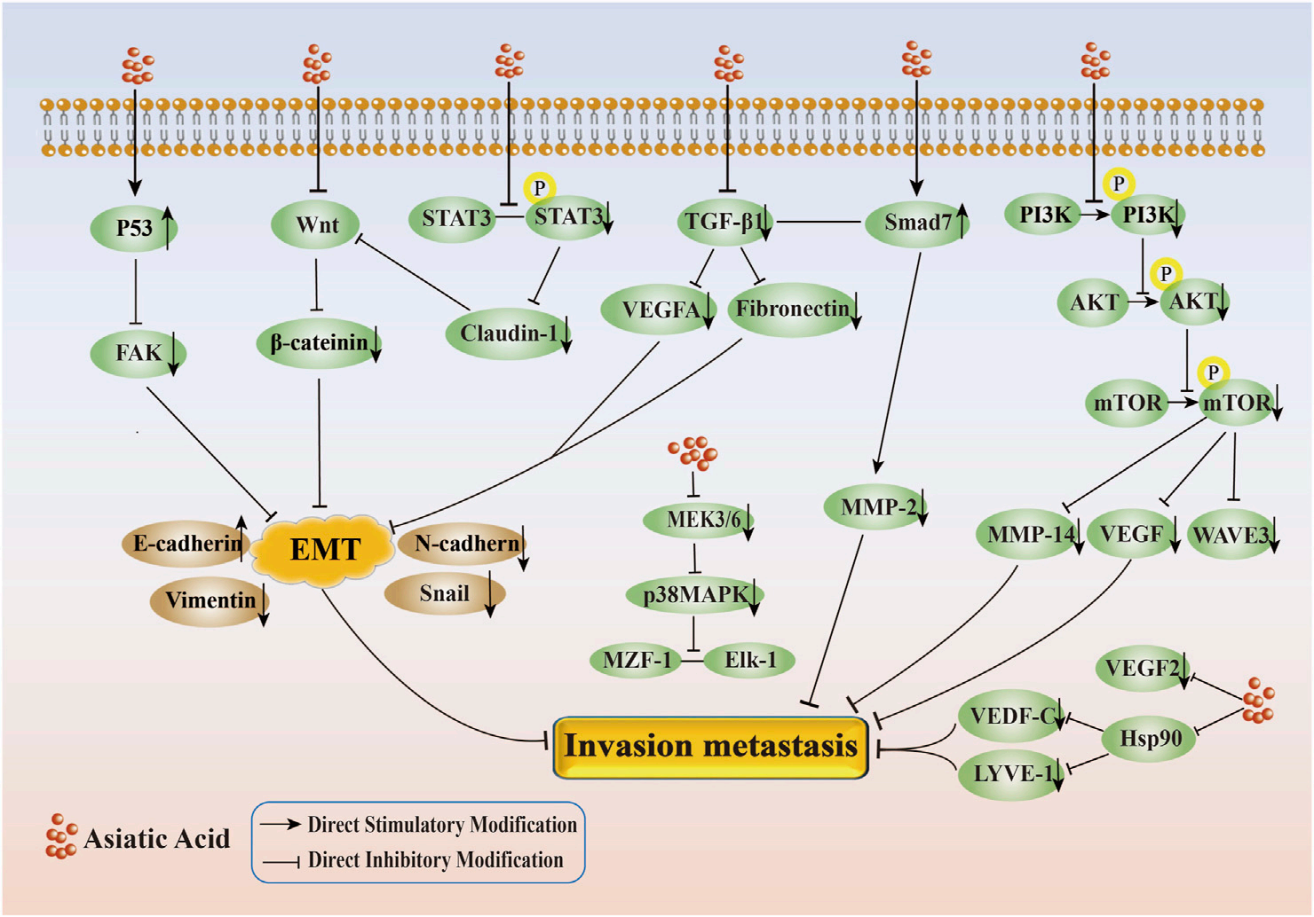
Mechanism of inhibition of tumor cell invasion and metastasis by Asiatic Acid. Asiatic Acid mainly regulates the changes of EMT related markers by inhibiting the Wnt/β - catenin axis and TGF-β axis, upregulating p53 and downregulating FAK, and inhibiting the expression of downstream target proteins by suppressing the PI3K/AKT, MEK3/6-p38/MAPK, and VEGF/VEGFR2 signaling pathways to suppress invasion and metastasis.

In conclusion, AA effectively inhibited the migration and invasion of tumor cells, mainly by inhibiting Wnt/β-catenin, TGF-β, PI3K/AKT, VEGF/VEGFR2, p38MAPK and other signaling pathways, upregulating p53 and downregulating the expression of proteins such as FAK, Hsp90, LYVE-1, and VEGF-C(Its specific mechanism of suppressing metastasis and invasion is shown in Figure 3).

## 5 Regulation autophagy in tumor cells by AA

Autophagy has been implicated in both development and progression of tumors, according to previous research, currently, there is a widespread belief that autophagy inhibits tumorigenesis. The complex role of autophagy in cancer appears to depend on tumor stage, particular oncogenic mutations, and microenvironment of tumor cells (Debnath et al., 2023).

According to a study by Li et al. (2015), Caspase-3 activation, inhibition of AKT expression, and reduction of mitochondrial membrane potential were observed in T98G glioma cells upon treatment with AA. Additionally, it decreased the expression of Beclin1, leading to the deactivation of the autophagy-related gene ATG protein. As a result, this inhibited the formation of autophagy precursor structures (PAS). Simultaneously, the LC3-II-PE ubiquitin-like protein system experienced a reduction in its effect, leading to a decrease in LC3-II levels and block formation of autophagosome, which results in inhibiting cell autophagy and promoting apoptosis.

P62 is the autophage adapter protein between the autophagosome and the substrate, and its levels are found to be inversely related to the autophagic activity (Zhao et al., 2018). Li (2021) identified several effects of AA on SMMC-7721 cells: a significant increase in the ROS, a decrease in autophagy protein P62 expression, an increase in the ratio of LC3-II/LC3-I, a decrease in Bcl-2 and an increase in the Bax, Caspase-3 and Cyt C expression in SMMC-7721 cells. Based on the results obtained from *in vitro and in vivo* studies, it could be inferred that AA possesses the capability to stimulate cell autophagy by triggering the accumulation of ROS, leading to the promotion of apoptosis and inhibition of proliferation of hepatocellular carcinoma cells. Geng et al. (2015) observed that the total protein content of LC3 in colon cancer HCT116 cells increased as the concentration of AA increased. Additionally, the expression of the active form of LC3-II increased significantly. However, the phosphorylation levels of p-mTOR and p-4EBP1 decreased over time, suggesting that autophagy is triggered by AA through the suppression of the mTOR-4EBP1 pathway, resulting in a considerable reduction of HCT116 cell proliferation.

**FIGURE 4:**
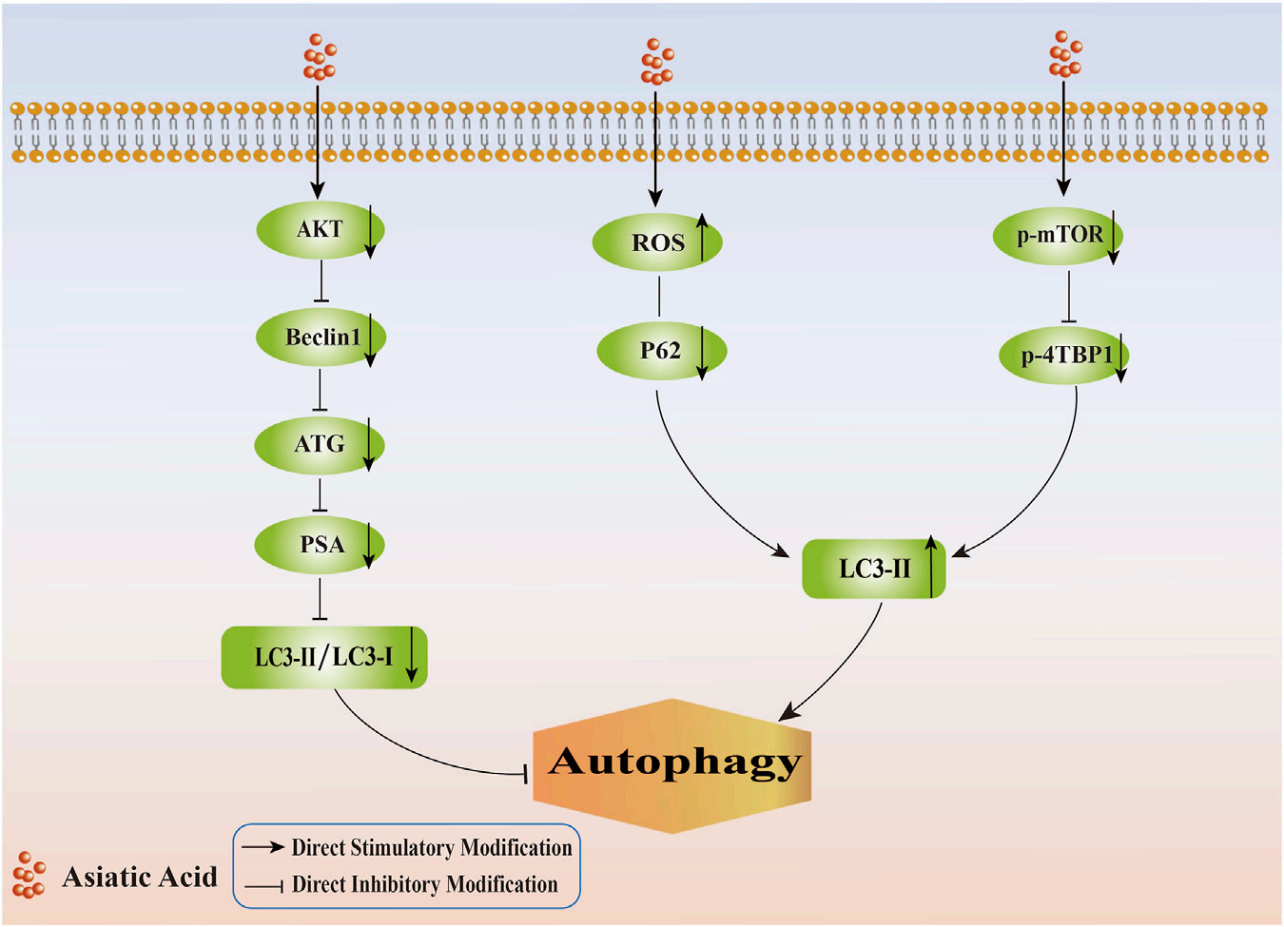
Mechanism of regulating tumor cell autophagy by Asiatic Acid. Asiatic Acid reduces the expression of AKT, Beclin1, ATG, PSA, and decrease the LC3II/LC3I ratio to inhibit autophagy. Asiatic Acid reduces autophagy by promoting ROS levels, reducing P62 protein, inhibiting the mTOR-4EBP1 pathway, and ultimately increasing the expression of LC3-II.

It appeared, based on the information provided, that AA inhibited the formation of autophagy, thereby promoting apoptosis in tumor cells, and could induce autophagy initiation, thereby inhibiting the proliferation of tumor cells (Its specific mechanism of regulating autophagy in tumor cells is shown in Figure 4). Since autophagy served as a double-edged sword, autophagy had intricate effects and mechanisms of action on tumor cells. Hence, there is a need for more investigation into the mechanisms by which AA regulates autophagy to promote the apoptosis of tumor cells and inhibit proliferation.

## 6 Improvment the sensitivity to chemotherapeutic drugs by AA

Drug resistance is a significant obstacle in the treatment of malignant tumors, although chemotherapeutic drugs have a better therapeutic effect as the first-line drugs for malignant tumors, their clinical application is often restricted due to the development of drug resistance, which is an issue that requires immediate attention and resolution (Fang et al., 2018).

Cheng et al. (2022) observed that AA had the ability to decrease the drug resistance of cisplatin-resistant human lung cancer A549/DDP cells. The observed impact is likely due to AA’s ability to downregulate metastasis associated lung adenocarcinoma transcript 1 (MALAT1), upregulate the expression of miR-1297, promote the degradation of p300, and reduce the nuclear translocation of β-catenind. It could reduce the expression level of multidrug resistance protein 1 (MDR1), make A549/DDP cells re-sensitive to DDP, and promote the apoptosis of A549/DDP cells. Liu et al. (2020) performed a research which found that AA significantly decreased the survival rate of DDP-resistant nasopharyngeal carcinoma NPC cells, induced an increase in Bax expression, and an upregulation of Caspase3, Caspase8, and Caspase 9. Additional, the phosphorylation of p38 and ERK1/2 pathways facilitated the expression of caspase-9. Therefore, it was shown that AA induced apoptosis in DDP-resistant NPC cells through a significant increase in p38 phosphorylation and a decrease in ERK phosphorylation.

According to the study conducted by Zhu et al. (2021), it was reported that AA had the ability to prevent the growth of doxorubicin (DOX)-resistant breast cancer MCF-7 cells. AA induced the apoptosis of MCF-7 cells through ROS generation, ATP content reduction, P-glycoprotein (P-gp) function improvement and adaptive immune balance. Additionally, AA activated the AMPK and NF-κB indirect transcription pathways to reverse DOX resistance. Additionally, the impact of AA on the immune checkpoint of cancer cells was investigated in this study, which revealed that AA downregulated the protein expression of PD-L1, thereby improving the resistance to DOX. Zhang T. et al. (2024) found that AA inhibited the proliferation of human chronic myeloid leukemia (K562/ADR) cells resistant to doxorubicin in a concentration dependent manner, reversing the cell’s resistance to ADR. By downregulating the expression of multidrug resistance protein-1 (MRP1) and P-gp, the efflux of chemotherapy drugs was reduced and the concentration of intracellular ADR is increased. The mechanism of reversing drug resistance was to inhibit the activation of the Wnt/β-catenin signaling pathway, thereby increasing the sensitivity of K562/ADR cells to ADR. These studies suggested that AA could potentially be used as a drug to overcome breast cancer cells resistance.

Chen et al. (2022b) identified a synergistic effect between AA and Oxaliplatin (OXP) in which AA enhanced the inhibitory effect of OXP against colon cancer HCT116 cell proliferation. Additionally, it also promoted apoptosis, autophagy, and pyroptosis in these cells. Its synergistic effect might be related to downregulating the expression of p62, upregulating the expressions of Cleaved Caspase-3, membrane perforation protein (GSDME), Beclin-1, LC3-II/LC3-I, and the ratio of Bax/Bcl2. Hence, the sensitization effect of AA on OXP exhibited the characteristics of having multiple targets and multiple pathways.

In addition, a study conducted by Chen X. C. et al. (2023) demonstrated that nephrotoxicity is the primary adverse effect seen in the clinical usage of DDP for treating solid tumors, and that long-term and low-dose administration of DDP might lead to the development of kidney fibrosis and inflammation. This study found that AA had a protective effect on chronic kidney impairment caused by long-term use of DDP and enhanced the anti-tumor efficacy of DDP. AA protects renal tubular epithelial cells against DDP-induced damage by restoring the equilibrium of Smad7/Smad3, and by promoting the protective autophagy-lysosomal pathway mediated by transcription factor EB. Overall, AA had a significant effect in reducing kidney damage, inflammation, and fibrosis produced by long-term DDP-injection in mice with tumors.

**FIGURE 5:**
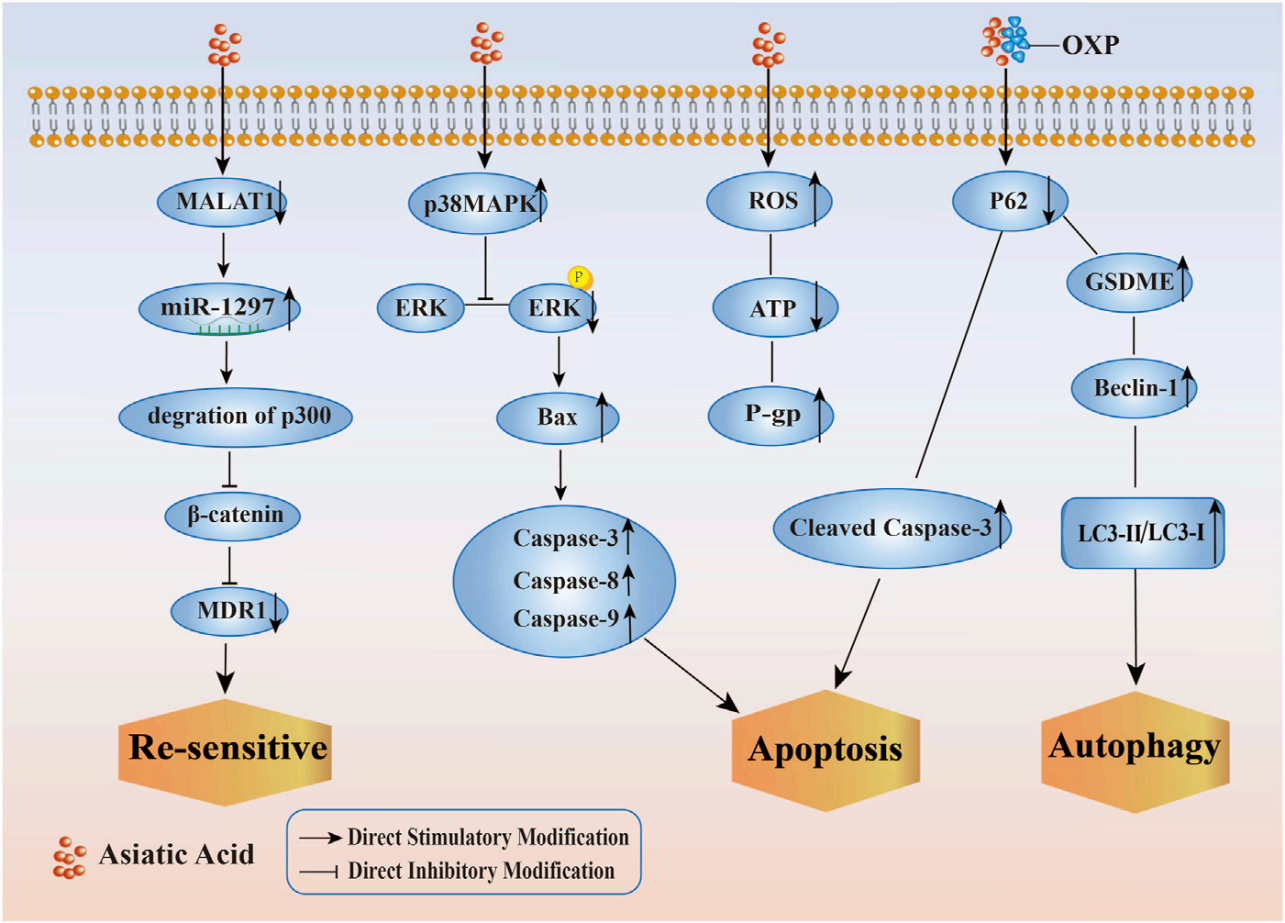
Mechanism of improving the sensitivity of tumor cells to chemotherapy and radiotherapy by Asiatic Acid. Asiatic Acid downregulates MALAT1, thereby reducing the expression of MDR1 and making drug-resistant tumor cells re sensitive; Asiatic Acid increases p38MAPK and decreases ERK pathway to regulate Caspase family and induce apoptosis; Asiatic Acid triggers the generation of reactive oxygen species, improves P-gp function and induces apoptosis; Asiatic Acid downregulates P62 and upregulates GSDME, Beclin-1, LC3-II/LC3-I to promote autophagy.

Chemotherapy resistance is a common phenomenon that occurs after chemotherapy and is a challenge in tumor treatment, in order to solve the adverse side effects and drug resistance of chemotherapy drugs, researchers have investigated the molecular effects of AA on multi-drug resistant cancer cell lines, including re-sensitivity to DDP, induction of apoptosis, reversal of DOX resistance, synergistic enhancement of sensitivity to OXP, and protection against kidney damage caused by DDP. (Its specific mechanism of improving the sensitivity of tumor cells to chemotherapy and radiotherapy by Asiatic Acid is shown in Figure 5). These studies demonstratied that AA enhanced the susceptibility of tumor cells to chemotherapy drugs and provided a foundation for the implementation of a therapeutic approach to treating tumors using a combination of traditional Chinese medicine and Western medicine. Therefore, AA is expected to become another option for patients with chemotherapy resistant tumors, as it has good anti-cancer effects and less toxicity.

## 7 Conclusion and outlook

Above all, AA had the capacity to inhibit tumor growth via multiple pathways and multiple targets. AA had high potential for development, but its research in the treatment of tumors is still not deep and comprehensive enough. In clinical practice, AA is mainly used for the treatment of surgical diseases such as skin wounds, chronic ulcers, delayed wound healing, surgical injuries, etc. However, most of the current research on AA treatment of tumors is still limited to animal experiments.

In addition, the low solubility, low bioavailability, and difficulty in crossing the blood-brain barrier of AA still limit its practical clinical trials. To address this issue, further research and development of AA derivatives and their new formulations are needed, and more detailed and comprehensive studies are needed to elucidate the molecular mechanisms and pharmacological activities of AA and its derivatives, in order to provide theoretical basis for establishing them as ideal therapeutic drugs for tumors.

Researchers have recently developed a range of well-designed derivatives of AA by modifying its structure at ring A, C-23 and C-28 carboxyl groups aiming to improve the anti-tumor efficacy of AA. In addition, significant advancements have been achieved in the use of carrier-based drug delivery systems for the anti-tumor treatment of AA. It has been reported that AA may specifically target and accumulate at the tumor site after loading into poly lactic-co-glycolic acid (PLGA) nanoparticle (AA-PLGA NPs), leading to an apoptosis-promoting effect. AA demonstrates potent anti-liver cancer properties and inhibits the process of EMT by using exosomes derived from Hepatocellular Carcinoma as a delivery system. DOX-AALip, a liposome loading with AA and adriamycin, exhibits enhanced accumulation in both the primary tumor and lung metastasis *in vivo*. The above investigations have conclusively shown that the effectiveness of AA, when administered using a drug delivery system, is superior to that of free AA. Therefore, it has been found that it has a greater efficiency in targeting tumors (Cunha et al., 2021; Ramalho et al., 2022; Chen et al., 2021; Zhang Y. et al., 2024). However, various kinds of tumors may need modifying the characteristics of different carriers or regulating the proportion of AA in order to achieve the highest drug delivery efficiency.

Moreover, the overexploitation of wild resources of *centella asiatica*, which is a source of AA, has attracted attention due to its unique medicinal value and rich bioactive components. The extraction, separation, identification, and structural modification of AA from *centella asiatica* are time-consuming, laborious, and technically challenging. Therefore, new extraction and separation technologies as well as efficient and environmentally friendly synthesis methods should be developed to reduce drug development time and costs. At the same time, investment in research on biosynthetic pathways should be increased to achieve large-scale production through synthetic biology, thereby solving the problem of sustainable access to natural resources and reducing dependence on natural resources.

In summary, as a highly promising cancer prevention and treatment drug, AA deserves further exploration of its pharmacological effects and mechanisms, in order to better utilize the advantages of traditional Chinese medicine and provide more choices for the treatment of tumors.

## Glossary

AA: Asiatic Acid
mTOR: mammalian target of rapamycin
p70S6K: 70 kDa Ribosomal Protein S6 Kinase 2
STAT3: signal transducer and activator of transcription 3
AKT: protein kinase B
AMPK: Adenosine 5‘-monophosphate (AMP)-activated protein kinase
JAK2: Janus kinase 2
JNK: c-Jun N-terminal kinase
MAPK: mitogen-activated protein kinase
ERK: extracellular signal-regulated kinase
Bcl-2: B-cell lymphoma-2
Bax: BCL2, associated X protein
Bcl-xL: B-cell lymphoma-XL
Cyt-C: cytochrome-c
PARP: poly, ADP-ribose polymerase
NF-κB: nuclear factor kappa-B
PI3K: Phosphoinositide-3 kinase
Grp78: glucose regulated protein 78kD
MZF-1: myeloid zinc finger 1
Elk-1: Phospho-ELk1 (Ser383)
HUVECs: Human Umbilical Vein Endothelial Cells
FAK: Focal Adhesion Kinase
GSH: Glutathione, Reduced
VEGF: Vascular endothelial growth factor
IRE1α: Immunoglobulin-Regulated Enhancer 1α
SP1: specifity protein 1
ROS: reactive oxygen species
EMT: epithelial–mesenchymal transition
Hsp90: Heat shock protein 90
LYVE-1: Lymphatic Vessel Endothelial Receptor-1
MMPs: Matrix metalloproteinases
P-gp: P-glycoprotein
PD-L1: programmed cell death-Ligand 1
OXP: Oxaliplatin
DOX: Doxorubicin
DDP: Cisplatin
